# Effects of Light Spectrum on Growth, Retinal Morphology, and Clock Gene Expression Patterns in *Takifugu rubripes* Larvae

**DOI:** 10.3390/biology15110870

**Published:** 2026-05-31

**Authors:** Zhen Yuan, Qi Liu, Yumeng Wu, Xuelian Luo, Pengfei Shen, Yuqing Xia, Hongwei Yan, Ying Liu

**Affiliations:** 1 College of Life Sciences, Liaoning Normal University, Dalian 116029, China; 2 Key Laboratory of Environment Controlled Aquaculture, Dalian Ocean University, Ministry of Education, Dalian 116023, China; 3 College of Marine Science and Environment Engineering, Dalian Ocean University, Dalian 116023, China; 4 College of Fisheries and Life Science, Dalian Ocean University, Dalian 116023, China; 5 College of Biosystems Engineering and Food Science, Zhejiang University, Hangzhou 310058, China

**Keywords:** circadian rhythm, growth performance, light spectrum, *Takifugu rubripes*, visual development, gene expression

## Abstract

Light exhibits a significant influence on aquatic organisms. This study assessed the effects of five light spectra (blue, red, green, yellow, and full-spectrum white) on the growth and survival of *Takifugu rubripes* larvae. The results showed that the wet weight, body length, and specific growth rate (SGR) of fugu larvae in the green and yellow light spectra groups were significantly better than those in the other groups. The qPCR results showed that growth hormone (*gh*) expression was significantly higher in larvae from the yellow light spectrum group. There was a significant increase in mRNA expression of *trypsinogen* in the yellow light spectrum group and pepsinogen in the green light spectrum group compared with the other groups, indicating that light regimes may help regulate endocrine function and digestive efficiency. The blue light spectrum significantly decreased the thickness of the photoreceptor layer (PRos/is), accompanied by increased expression of the apoptosis marker caspase3, which may be related to decreased expression of melanin-concentrating hormone receptors (*mchr1/2*). Analysis of clock genes (such as *per3*, *per1a*, and *cry2*) revealed disrupted expression patterns under blue light, particularly at Zeitgeber time 12 (lights-off), indicating interference with circadian regulation. Our study indicated that optimizing lighting by prioritizing the green or yellow light spectrum can enhance growth efficiency and minimizing prolonged exposure to blue light is essential for retinal health during *T. rubripes* aquaculture.

## Simple Summary

We evaluated the effects of five light spectra (blue, red, green, yellow, and full-spectrum white) on growth, visual development, and gene expression in *Takifugu rubripes* larvae. Green and yellow light spectra promoted growth, elevated growth hormone expression, and enhanced digestive gene expression. In contrast, blue light induced retinal thinning, increased apoptosis-related gene expression, and disrupted clock gene rhythms. These results establish green and yellow light spectra as optimal for *T. rubripes* larviculture and highlight the adverse physiological consequences of prolonged blue light exposure.

## 1. Introduction

Light as a fundamental environmental factor, exhibits a significant influence on aquatic organisms. The effect is mediated by specific characteristics: quality (spectrum), quantity (intensity), and periodicity (photoperiod) [1]. Light manipulation has been widely applied to optimize the survival rates and growth performance of fish, consequently leading to a reduction in aquaculture costs [2]. However, it should be noted that the physical properties of water differ significantly from those of the surrounding air environment. As the concentration of suspended particulate matter or water depth varies, the spectral composition of ambient light also shifts in natural aquatic environments. Short-wavelength radiation (violet and blue) is scattered more strongly in shallow or turbid waters, while long-wavelength radiation (red and orange) is scattered less but absorbed more rapidly by water molecules and dissolved organic matter, depending on water clarity [3,4,5]. Therefore, fish have evolved preferences for different light environments. Even during different stages of development, these preferences are obviously distinct [6]. Previous studies indicated that different light spectra could affect fish survival [7], growth performance [8], feed intake [9], reproduction [10], and behavior, including individual hierarchical status [11], aggressive behavior [12,13], and search behavior [14].

LED lights offer significant advantages for aquaculture, including energy conservation, environmental friendliness, and high photoelectric conversion efficiency. Most importantly, they enable precise and independent control over key light parameters, such as spectrum, intensity, and photoperiod. This allows tailored light regimes to meet specific production and physiological requirements [15]. Consequently, there has been a gradual increase in the utilization of LED light sources in aquaculture [16]. For example, the blue light spectrum can raise the growth performance of *Scopillatus maximus* and *Carassius auratus* [17], increase the survival of *Dicentrarchus labrax* [18], and enhance the immunity of *Amphprion melanopus* [19]. However, *Oncorhynchus mykiss* exhibited the most significant body weight and length under yellow light spectrum conditions [20], and the yellow light spectrum has been shown to promote the growth of *Trichopodus leerii* [21]. In *Gadus morhua*, the green light spectrum was shown to promote growth [18]. By promoting the mRNA expression of growth hormone (*gh*), mean corpuscular hemoglobin (*mch*), and neuropeptide y (*npy*) under green light spectrum conditions, the body length, wet weight, specific growth rate (SGR), and survival rate of *Micropterus salmoides* larvae were significantly elevated [22]. The green light spectrum can effectively control the oxidative stress of *C. auratus* and reduce the production of free radicals [23]. Compared with other spectrums, *O. mykiss* has the highest growth and survival rate under red light conditions [24]. The SGR and feed conversion ratio of *Haliotis discus hannai* under red and yellow light spectra were significantly higher than those under the blue light spectrum [25]. The above studies have demonstrated that, from egg hatching to fish growth, it is crucial to select optimal light conditions, with specific spectral compositions, at the right time for fish culture. In addition, the maximum growth rate of fish is closely related to their digestive capacity [26]. The essential digestive enzymes contain pepsin, which is secreted by the gastric mucosa, and trypsin, which is secreted by the pancreas and present in the intestine [27]. As demonstrated by Durigon, trypsin has been identified as an enzyme exhibiting rhythmic activity in *O. mykiss* [28]. Research indicates that *pepsinogen* (precursor of pepsin) expression directly influences gastric functional maturation in *Synchiropus splendidus* larvae [29]. Growth enhancement in *Sparidentex hasta* larvae relies on pepsin’s proteolytic processing [30]. However, research on how the environment regulates digestive factors is lacking in teleost [31]. Additionally, whether light spectrum can affect the digestive ability have not been reported previously.

Research has demonstrated that the plasticity of fish retina development is influenced by varying light conditions [32]. Spectrum compositions can affect retinal structure and function by altering photoreceptor sensitivity and opsin expression [33]. For example, Wagner (2005) found that the structure of the inner layer of the retina underwent changes in response to alterations in the spectrum following a period of 1~2 years of exposure to monochromatic light conditions in *Aequidens pulcher* [34]. Although fish larvae can also use non-visual cues to locate prey at a distant location, vision still plays an indispensable role in their catch activities [35]. In aquaculture, even though the bait density in the pond is higher than that in the natural habitat of fish, the prey still needs to enter the visual range of young fish to be found and preyed on. Hence, vision-optimized lighting regimes improve from larval (20 dah) to early juvenile (80 dah) stages, enhancing foraging efficacy and growth performance. Based on the high plasticity of the fish retina, it is important to study the occurrence and development of the retina structure of fish under different light conditions to understand their physiological activities, ecological adaptability, and other aspects.

In aquaculture, the maintenance of robust circadian rhythms is crucial for optimizing feed efficiency, growth performance, and overall fish welfare. Circadian rhythms are controlled by clock genes and regulate growth, development, and physiological functions in organisms [36,37]. Period gene (*per1*, *per3*) and cryptochrome 2 (*cry2*) are known representative clock genes [38]. *Per1* is a crucial component of the molecular oscillation system that regulates biological rhythms and participates in physiological and biochemical activities. The period gene family is the leading member of the physiological rhythm regulation genes, in which period 1 (*per1a*) plays a core role. *Per3* is expressed in the suprachiasmatic nucleus in a circadian pattern [39]. CRY2 is a key regulator of the circadian clock mechanism [40]. In fish, light affects the regulation of the circadian rhythm by influencing the expression of genes like *per* and *cry* [41,42]. In *Danio rerio* larvae, it has been reported that the spectrum affects clock gene (*per* and *cry*) expression and activity rhythms. Expression levels of arylalkylamine N-acetyltransferase and melatonin receptors were significantly higher under green and blue light spectra than under full-spectrum white light spectrum [43].

The *Takifugu rubripes* belongs to the order Tetraodontiformes, family Tetraodontidae, and genus Takifugu. Due to its high nutritional value and economic potential, *T. rubripes* has become a significant economic importance to aquaculture in China, Japan, and South Korea [44]. In 2020, the aquaculture production of *T. rubripes* reached over 17,473 t [45]. To advance *T. rubripes* aquaculture, it is imperative to elucidate how environmental factors influence this species, thereby enabling the optimization of rearing conditions and the enhancement of its commercial value. Previous studies have found that the exposure of *T. rubripes* to a 20 L:4 D photoperiod and 250~500 mW/m^2^/s light intensity benefits their growth and development. Green light activates GH/IGF-I axis gene expression and suppresses apoptosis in *T. rubripes* [22]. Full-spectrum white light and blue light significantly increase the amino acid content in the muscle of *T. rubripes* [46]. However, the actual effects of the spectrum on the growth, retinal development, and circadian rhythm of *T. rubripes* larvae remain unclear.

The purpose of this study was to explore the effects of different spectra on the growth, survival, visual development, and rhythm of *T. rubripes* and to reveal the expression of clock genes, as well as the activity of digestive enzymes, in order to optimize culture conditions for *T. rubripes* larvae. Given that *T. rubripes* inhabits coastal and estuarine environments, where water turbidity often attenuates shorter wavelengths and shifts the available spectra toward green–yellow regions [3], we propose the following hypotheses: (1) green and yellow light spectra (525–595 nm), as the wavelengths most closely matching the natural photic environment of this species, will produce the most favorable outcomes for larval growth performance, digestive function, and visual development; (2) conversely, the short-wavelength blue light spectrum (450–455 nm), which has limited penetration in the turbid natural habitats of this species and has been associated with retinal photodamage in other teleosts, will adversely affect retinal structural integrity and circadian clock entrainment. Our results provide a theoretical basis for applying LED lights in the aquaculture production of *T. rubripes*.

## 2. Materials and Methods

### 2.1. Eggs Incubation and Larvae Rearing

During the egg incubation period, fertilized eggs were randomly transferred to five 100 L (62 cm height) tanks, each containing 25 g of eggs. The fertilized eggs were initially reared under continuous LED light sources with five different spectra, at a light intensity of 0.5 W/m^2^/s, separately. Salinity, temperature, dissolved oxygen, and pH were monitored daily. The fish were raised at approximately 19.5–21.5 °C and salinity at 32 for 60 days (from 20 to 80 dah). The pH was maintained at 7–8, and the oxygen level was maintained at 6–8 mg/L. Ammonia and nitrites were measured weekly, and the mean values were always <0.2 and <0.05 mg L^−1^, respectively.

After the eggs hatch, all the fish under the per light spectrum were transferred to three 100 L tanks, separately, for subsequent larval culture. The larvae were fed *Rotifer*, *Artemia*, and commercial feed during the culture period, depending on the conditions for larval development. *Rotifers* were separately provided at 4–10 day after hatching (dah). *Rotifers* and *Artemia* were simultaneously offered at 11–12 dah. *Artemia* was separately provided at 13–22 dah. Commercial feed and *Artemia* were alternately offered at 23–32 dah. Commercial feed was given at 33–80 dah. The fish were fed to satiation approximately six times a day. At 20 dah, larvae with a body length ± standard deviation (SD) of 8.17 ± 0.9 mm were raised at an initial density of 750 larvae per tank before the experiment began. Subsequently, this experiment was conducted from 20 dah to 80 dah.

### 2.2. Experimental Design

Five lighting treatments were tested, each with three replicates (a total of 15 experimental units), including blue (450~455 nm), red (625~630 nm), green (525~530 nm), yellow (590~595 nm), and full-spectrum white (400~780 nm), all with an intensity of 0.5 W/m^2^/s. The photoperiod was changed to 12 L:12 D after egg hatching. A lighting analyzer (PLA-20, Hangzhou, China) was used to measure both the intensity and spectral composition of light. Each tank was separated by an opaque grey screen to block external light in order to minimize the influence of background light on the experiment. The light intensity of four points was averaged and calibrated at 1 cm above the water surface of each barrel using a lighting analyzer (PLA-20, Hangzhou, China) at 8:30 am daily. All experiments in this study were conducted in accordance with the guidelines for animal protection and utilization at Dalian Ocean University.

### 2.3. Growth and Survival

At 20, 60, and 80 dah, 30 larvae per tank were randomly selected for sampling. The total length, body length, eye diameter, and wet weight per fish were recorded. The morphology measurements were performed using a microscope (S8Apo, Leica, Wetzlar, Germany) equipped with a camera (sCCD01, ToupTek, Zhejiang, China).

The SGR was calculated using the following equation:

(1) $$SGR=\frac{\left(\ln final\;weight\right)-\left(\ln initial\;weight\right)}{\mathrm{Number}\;\mathrm{of}\;\mathrm{days}}\times 100\%$$

The survival rate (SR, %) was calculated using the following equation:

(2) $$SR(\%)=\left[\frac{\left(n0-d1\right)}{n0}\frac{\left(n0-d1-s1-d2\right)}{\left(n0-d1-s1\right)}\right]\times 100\%$$

where *n*0 is the number of fish at the beginning;

*d*1 is the quantity of dead fish during the initial phase (prior to the initial sampling);

*s*1 is the number of fish sampled for the first time;

*d*2 is the number of dead fish in the second stage (prior to the second sampling, but following the first sampling).

### 2.4. Retinal Histology and Morphological Analysis

At the end of the experiment, the heads of five randomly selected larvae in each tank were immersed in 4% paraformaldehyde for 24~28 h and then transferred to 70% ethanol. Prior to embedding, the tissues were dehydrated using a graded ethanol series: 80% (1.5 h), 90% (1 h), 95% (1 h), and two changes of 100% ethanol (0.5 h each). Subsequently, the tissues were cleared in xylene and embedded in paraffin. Next, 4 μm slices were prepared using a microtome, followed by routine histological staining with H&E. The sections were examined and imaged using a Leica DM4 B light microscope equipped with a Leica DFC7000 T digital camera (Leica Microsystems, Wetzlar, Germany). LAS X was used for retinal measurement (LAS_X_4.7.0, Rockville, MD, USA). The thickness of each layer, including the ganglion cell layer (GCL), optic fiber layer (OFL), outer nuclear layer (ONL), outer plexiform layer (OPL), inner nuclear layer (INL), inner plexiform layer (IPL), photoreceptor layer (PRos/is), and retinal pigment epithelium layer (RPE), was measured in μm. Additionally, the nuclear density of ONL, INL, and GCL was measured in cells per millimeter. The data from each tank were treated as an independent biological replicate in the statistical analysis.

### 2.5. RNA Extraction and qPCR

In order to quantify the expression of clock genes, the brains of larvae from each tank were dissected at four different time points at the end of the experiment. Each tank was considered a biological replicate, and 20 larvae were sampled from each tank. The time points were defined relative to Zeitgeber time (ZT). Zeitgeber time 0 (ZT 0, lights-on), ZT 6 (6 h after lights-on), ZT 12 (lights-off) and ZT 18 (6 h after lights-off). In addition, the intestines of larvae from each tank were dissected at the end of the experiment. Each tank was considered a biological replicate, and 20 larvae were sampled from each tank. All samples were quickly submerged in RNAlater^TM^ (Beyotime, Dalian, China) RNA stabilization reagent and subsequently preserved at −80 °C. RNA was extracted using the RNeasy Mini Kit (Qiagen, Hilden, Germany). An Agilent 2100 biological analyzer (Agilent 2100, Agilent, Folsom, CA, USA) and NanoDrop spectrophotometer (Thermo Fisher Scientific, Wilmington, DE, USA) were used to check the RNA concentration and integrity. The RNA integrity number (RIN) values of all samples used for qPCR analysis ranged from 8.2 to 9.5. The PrimeScript™ RT reagent kit with gDNA Eraser (Takara, Dalian, China) was used to obtain cDNA according to the manufacturer’s instructions. Then, we stored the cDNA products at 20 °C. Real-time quantitative PCR experiments were performed using LightCycler^®^ 96 (BBI, Roche, Germany) instrument. The primer sequences were designed using Primer 5 software (Primer Premier 5) (Table 1) [47]. According to the manufacturer’s instructions, the stability of two genes (*β-actin* and *efα1*) in *T. rubripes* was verified at the beginning of the experiment. The results revealed that *β-actin* was more stable. Therefore, *β-actin* was selected as the endogenous reference gene for normalizing all qPCR data. The total volume of the PCR reaction mixture for gene amplification was 20 μL, consisting of 32 ng cDNA template, 10 μL TB Green Premix Ex Taq II (Takara), and 0.8 μM of any gene-specific primer. RT-qPCR was performed in triplicate: 30 s at 95 °C; then 40 cycles at 95 °C for 5 s, 60 °C for 30 s; then the next cycle at 95 °C for 5 s; and finally hold at 50 °C for 30 s. All qPCR reactions were run in triplicate, and the relative gene expression levels were subsequently calculated using the 2^−ΔΔCT^ method [48]. The data from each tank were treated as an independent biological replicate in the statistical analysis.

### 2.6. Statistical Analysis

Data are expressed as the means ± SEM (*n* = 3 tanks per group). In order to investigate the statistical differences among treatment parameters, one-way analysis of variance (ANOVA) and Duncan’s test were used to analyze all the data. SPSS version 22.0 (Armonk, NY, USA) was used, and *p* < 0.05 indicated a significant difference. Origin 2017 (Hampton, MA, USA) was used to generate charts.

## 3. Results

### 3.1. Growth Performance, Survival, and Expression of Growth- and Digestion-Related Genes

The variations in wet weight, body length, total length, and eye diameter of *T. rubripes* reared under different light spectra are shown in Table 2. At 20 dah, the body length of larvae reared in the red (R), green (G), and yellow (Y) groups was significantly larger than that in blue (B) light spectrum group (*p* < 0.05). At 60 dah, the body length of larvae in the green group was significantly greater than that in blue and full-spectrum white (W) groups (*p* < 0.05). At the end of our experiment, it was obviously shown that the green, yellow, and full-spectrum white groups larvae had a significantly greater body length than that of larvae reared in the blue and red groups. At 80 dah, similar to body length, the total length of larvae reared in the green, yellow, and full-spectrum white groups was also significantly greater than that in the blue and red groups (*p* < 0.05). Additionally, at 20 dah, there was no significant variation in eye diameter across all treatments (*p* > 0.05). The eye diameter of the larvae in the full-spectrum white group was found to be significantly smaller than that of the other four groups at 60 dah (*p* < 0.05). In contrast, no significant difference in eye diameter was observed among the five treatments at 80 dah (*p* > 0.05). After the fertilized eggs hatched, at 20 dah, all the mean values of wet weight were lower than 0.022 g. However, the wet weight of larvae in the yellow light spectrum group was significantly higher than that of the other four groups (*p* < 0.05). At 60 dah, the wet weight of larvae in the green group was significantly higher than that of the other four groups (*p* < 0.05). At 80 dah, larvae reared in the green, yellow, and full-spectrum white light spectra groups showed significantly higher wet weight than that in other groups (*p* < 0.05) (Table 2).

**Figure 1:**
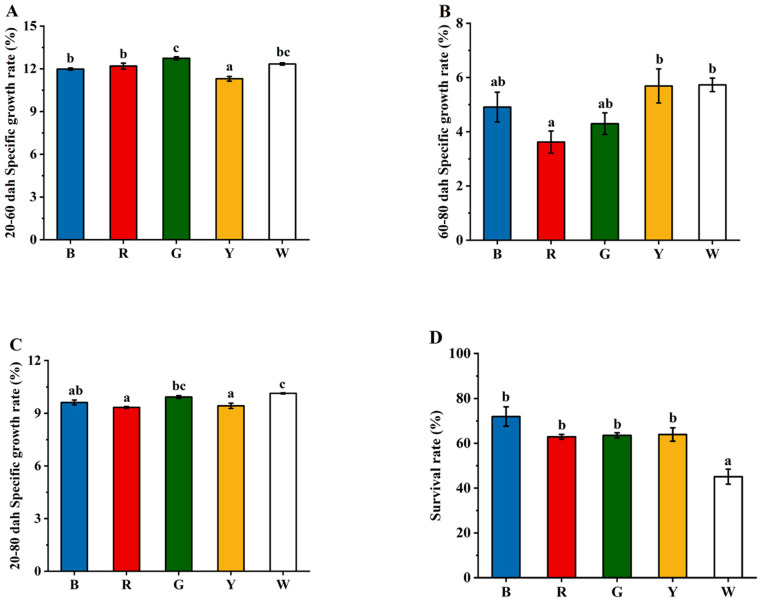
Specific growth rate (SGR) of *Takifugu rubripes* larvae reared under blue (B), red (R), green (G), yellow (Y), and full-spectrum white (W) light. SGR was calculated for the periods 20–60 dah (**A**), 60–80 dah (**B**), and 20–80 dah (**C**). The SR (**D**) of *T. rubripes* larvae reared under blue (B), red (R), green (G), yellow (Y), and full-spectrum white (W) light at 80 dah. Different lowercase letters indicate significant differences between each treatment group (one-way ANOVA, *p* < 0.05, n = 3 tanks per group). Bars sharing the same letter are not significantly different.

In the present study, the SGR of larvae was calculated from 20~60 dah, 60~80 dah, and 20~80 dah, separately (Figure 1A–C). At 20~60 dah, the SGR of larvae in the green light spectrum group was the greatest (10.31%) and significantly higher than that in the blue, red, and yellow light spectra groups (*p* < 0.05), but no significant difference was observed compared with the full-spectrum white group (*p* > 0.05) (Figure 1A). At the conclusion of the experiment, the survival rate in the full-spectrum white group was significantly lower in comparison to the other four groups (*p* < 0.05), whereas no significant difference was observed among the remaining groups (*p* > 0.05) (Figure 1D).

**Figure 2:**
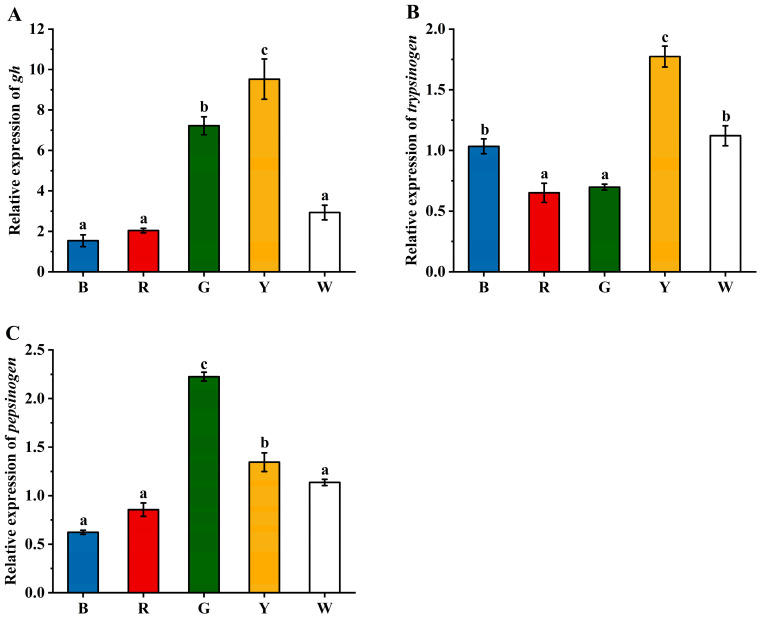
Effect of different spectrum on the *gh* (**A**), *trypsinogen* (**B**), and *pepsinogen* (**C**) expressions of *T. rubripes* larvae reared under blue (B), red (R), green (G), yellow (Y), and full-spectrum white (W) light at 80 dah. Different lowercase letters indicate significant differences between each treatment group (one-way ANOVA, *p* < 0.05, data are expressed as the means ± SEM, n = 3 tanks per group). Bars sharing the same letter are not significantly different.

At the end of the experiment, the expression levels of *gh*, *trypsinogen*, and *pepsinogen* in *T. rubripes* reared under different light treatments were illustrated in Figure 2A. Compared with other groups, *gh* expression in larvae from the green and yellow groups was substantially higher at 80 days (*p* < 0.05) (Figure 2A). Meanwhile, the expression of *trypsinogen* in larvae from the yellow group was also significantly higher than that of other groups (*p* < 0.05) (Figure 2B). Similar to the above results, *pepsinogen* expression in larvae was also significantly higher in the green and yellow groups compared with the other groups (*p* < 0.05) (Figure 2C).

### 3.2. Effect of Different Light Spectra on Visual Development and Vision-Related Gene Expression

**Figure 3:**
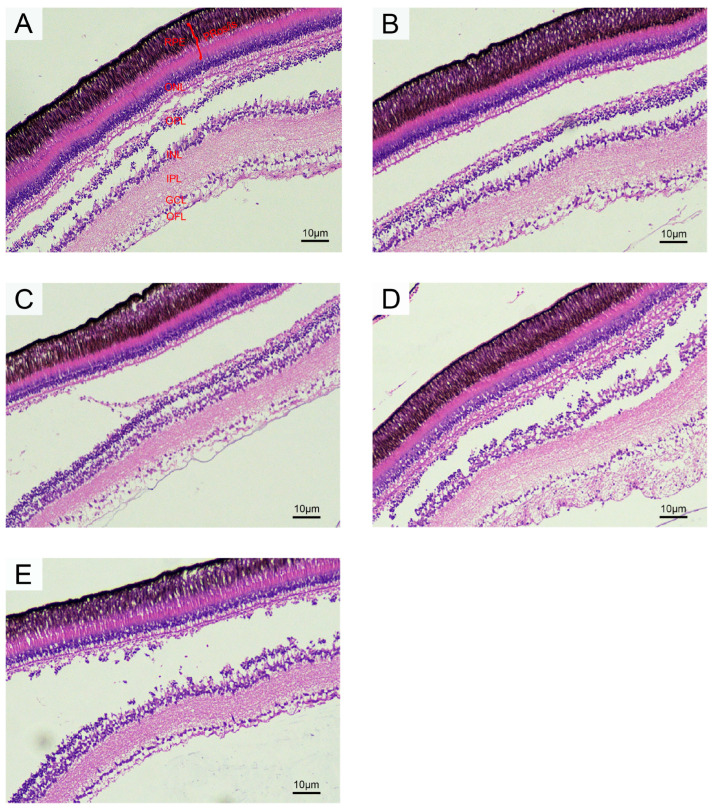
Histological structure of the retina of *T. rubripes* larvae reared under different light spectra at 80 dah. (**A**) Blue light spectrum group. (**B**) Red light spectrum group. (**C**) Green light spectrum group. (**D**) Yellow light spectrum group. (**E**) Full-spectrum white light group. The thickness of each layer, including the ganglion cell layer (GCL), optic fiber layer (OFL), outer nuclear layer (ONL), outer plexiform layer (OPL), inner nuclear layer (INL), inner plexiform layer (IPL), photoreceptor layer (PRos/is), and retinal pigment epithelium layer (RPE), was measured in μm. Additionally, the nuclear density of ONL, INL, and GCL was measured in cells per millimeter. Scale bar = 10 μm.

Histological analysis was conducted on the retinas of *T. rubripes* larvae raised under five different light spectra. The ten-layer structure of the retina was evident based on histological results (Figure 3). No significant differences were observed in the histological sections of the retinas under different light conditions (Figure 3). The thickness of each layer was measured and calculated. The nuclear density of ONL, INL, and GCL was measured in cells per millimeter.

**Figure 4:**
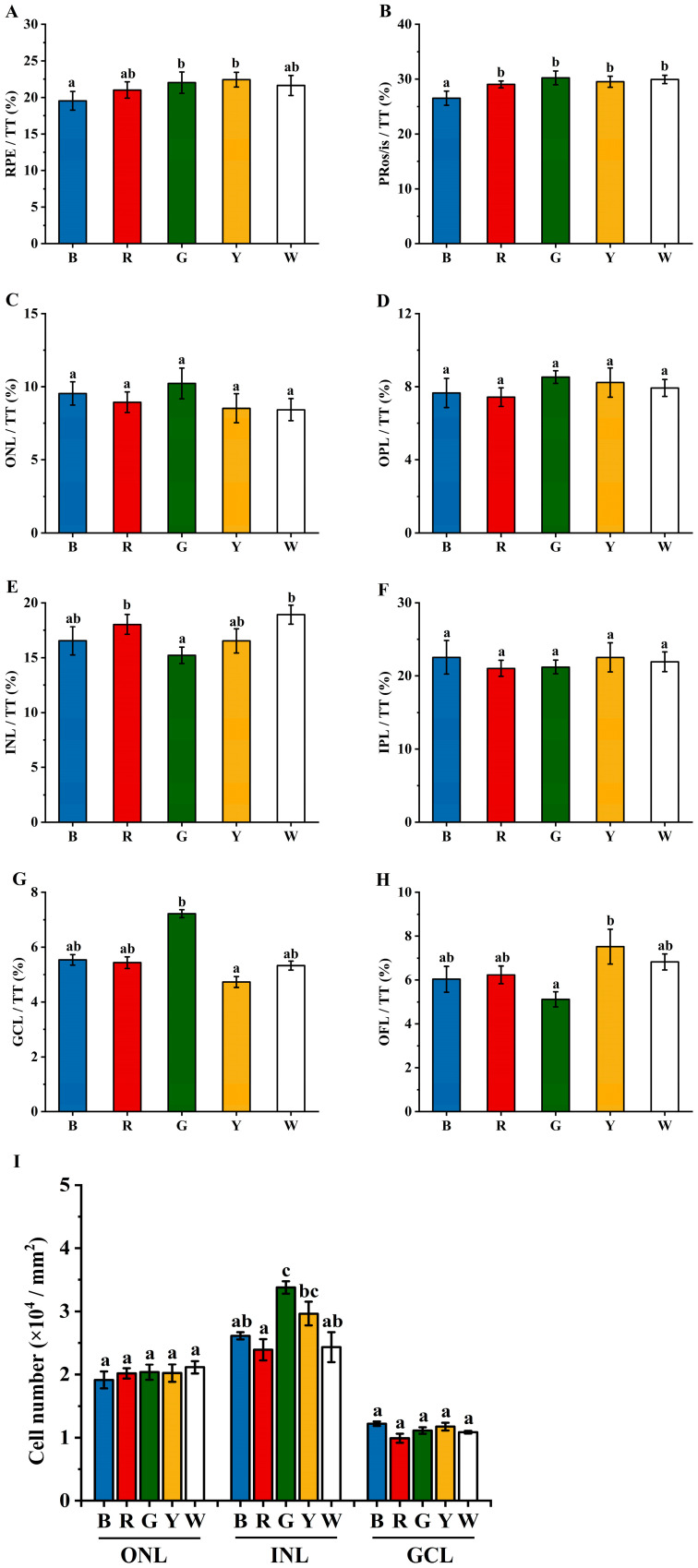
The ratio of the thickness of each retinal layer to total thickness in *T. rubripes* larvae (80 dah) reared under blue (B), red (R), green (G), yellow (Y), and full-spectrum white (W) light spectra ((**A**): RPE/TT, (**B**): PRos/is/TT, (**C**): ONL/TT, (**D**): OPL/TT, (**E**): INL/TT, (**F**): IPL/TT, (**G**): GCL/TT, (**H**): OFL/TT), as well as the nuclear density (individuals/mm^2^) of the outer nuclear layer (ONL), inner nuclear layer (INL), and ganglion cell layer (GCL) of larvae from different spectra (**I**). Different lowercase letters indicate significant differences between each treatment group (one-way ANOVA, *p* < 0.05, n = 3 tanks per group). Bars sharing the same letter are not significantly different.

It was found that different light spectra did not affect the total thickness of the retina (*p* > 0.05) (Figure 4). Additionally, the ratios of thickness of each retinal layer to the total thickness were calculated. The ratios of ONL/TT, OPL/TT, and IPL/TT did not show significant differences among light treatments (*p* > 0.05) (Figure 4C–F). Nevertheless, the ratio of PRE/TT in larvae reared in the blue group was found to be significantly lower than that observed in larvae reared in the green and yellow groups (*p* < 0.05) (Figure 4A). Furthermore, the ratio of PRos/is/TT in larvae reared in the blue group was found to be significantly lower in comparison to that observed in larvae subject to the other light spectra treatments (*p* < 0.05) (Figure 4B). The ratio of INL/TT in larvae exposed to the green light spectrum was found to be significantly lower than those exposed to red or full-spectrum white light (*p* < 0.05) (Figure 4E). Likewise, the ratio of GCL/TT under yellow light spectrum treatment was significantly lower than that under green light spectrum treatment (*p* < 0.05) (Figure 4G). Moreover, a statistically significant disparity was observed in the ratio of OFL/TT in larvae reared under green light spectrum treatment compared to larvae exposed to yellow light spectrum treatment (*p* < 0.05) (Figure 4H). Cell nucleus density in the ONL and GCL showed no significant differences among different light spectrum treatments (*p* > 0.05) (Figure 4C). However, the INL cell nucleus density of the retina in larvae reared under red light spectrum treatment was significantly lower than those exposed to green and yellow light spectrum treatments (*p* < 0.05) (Figure 4E).

**Figure 5:**
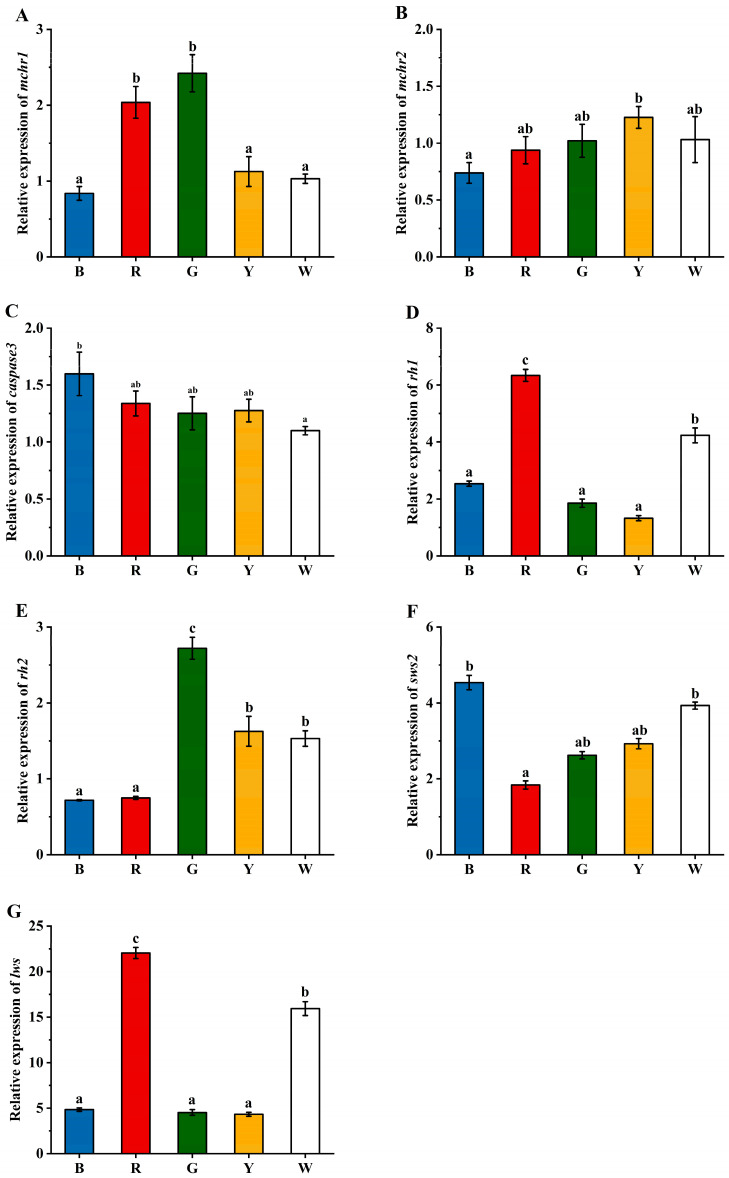
Effects of different light spectra on the expression of vision-related genes in *T. rubripes* larvae under blue (B), red (R), green (G), yellow (Y), and full-spectrum white (W) spectrum at 80 dah ((**A**): *mchr1*, (**B**): *mchr2*, (**C**): *caspase3*, (**D**): *rh1*, (**E**): *rh2*, (**F**): *sws2*, (**G**): *lws*). Different lowercase letters indicate significant differences between each treatment group (one-way ANOVA, *p* < 0.05). Data are expressed as the means ± SEM, with n = 3 tanks per group. Bars sharing the same letter are not significantly different.

In addition, mRNA expression changes of visual-related genes *mchr1*, *mchr2*, *caspase3*, *rh1*, *rh2*, *sws2*, and *lws* in eyes were measured in *T. rubripes*. The expression level of *mchr1* was significantly lower in the blue, yellow, and full-spectrum white light spectra groups compared to the green and red light spectra groups (*p* < 0.05) (Figure 5A). The expression level of *mchr2* was significantly lower in the blue light group compared to the yellow light group (*p* < 0.05) (Figure 5B). The expression level of *caspase3* was significantly higher in the blue light treatment group compared to the full-spectrum white light treatment group (*p* < 0.05) (Figure 5C). The expression level of *rh1*was significantly higher in the red light spectrum group compared to all other treatment groups (*p* < 0.05) (Figure 5D). In contrast to the green, yellow, and full-spectrum white light treatment groups, the blue light spectrum and red light spectrum treatment groups exhibited a significantly lower level of *rh2* expression (*p* < 0.05) (Figure 5E). Compared to the red light spectrum group, the expression level of *sws2* was significantly higher in the blue and full-spectrum white light groups (*p* < 0.05) (Figure 5F). The expression level of *lws* was significantly higher in the red light spectrum group compared to all other treatment groups (*p* < 0.05) (Figure 5G).

### 3.3. Effects of Different Light Spectra on the Circadian Rhythm of Clock Gene Expression in Brain

**Figure 6:**
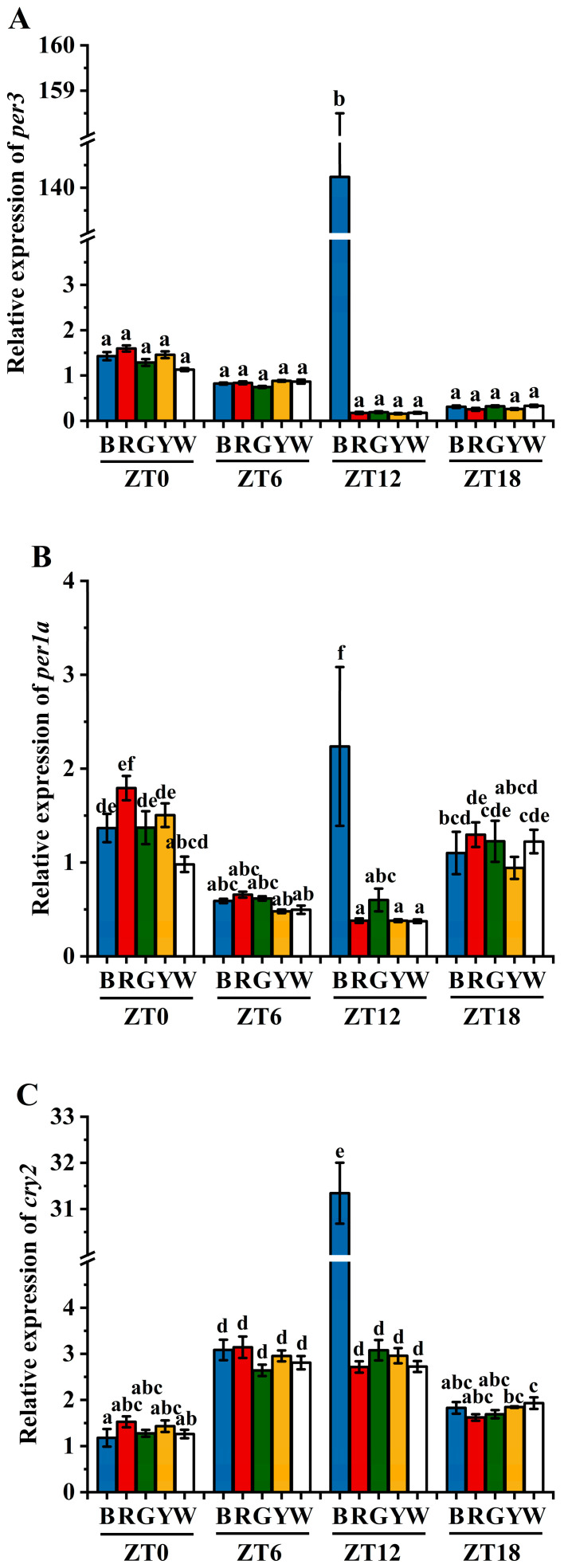
Circadian rhythm of *per3* (**A**), *per1a* (**B**), *cry2* (**C**) expression in the brain of *T. rubripes* larvae reared under blue (B), red (R), green (G), yellow (Y), and full-spectrum white (W) light at 80 dah. The columns represent *per3*, *per1a* and *cry2* expression (left *Y*-axis). Zeitgeber time 0 (ZT 0, lights-on), ZT 6 (6 h after lights-on), ZT 12 (lights-off), and ZT 18 (6 h after lights-off). Different lowercase letters indicate significant differences between each treatment group (one-way ANOVA, *p* < 0.05, data are expressed as the means ± SEM, n = 3 tanks per group). Bars sharing the same letter are not significantly different.

Figure 6 shows the effects of different light treatments on the circadian rhythm of clock gene expression in brain. The results revealed that the expression level of *per3* was significantly higher at ZT 12 under the blue light spectrum (*p* < 0.05). On the other hand, the expression trends of *per3* were similar when larvae were reared under red, green, yellow, and full-spectrum white light treatments. With the exception of the blue light spectrum group, it was evident that *per3* reached its peak at ZT 0, gradually decreased over time, reached its minimum at ZT 12, and then increased at ZT 18 (Figure 6A). Additionally, the expression levels of *per1a* and *cry2* were also the highest at ZT 12 under blue light spectrum and were significantly higher than those in other treatments (*p* < 0.05). The expression trend of *per1a* was almost similar to that of *per3*. With the exception of the blue light spectrum group, *per1a* expression peaked at ZT 0, decreased to ZT 12, and then increased again at ZT 18 (Figure 6B). In the red, green, yellow and full-spectrum white light groups, the expression levels of *cry2* gene initially increased from ZT 0 to ZT 6 and then decreased after ZT 12 (Figure 6C).

## 4. Discussion

### 4.1. Effects of Light Spectrum on Growth Performance and Digestive Function

In this study, larvae reared under green, yellow, and full-spectrum white light exhibited optimal growth, with no significant differences among the three groups. However, the survival rate in the full-spectrum white light group was significantly lower compared to the other groups. This study reveals spectrum-specific effects on *T. rubripes* larvae, with the optimal spectra being the green and yellow light spectra (525~595 nm). A similar phenomenon has also been observed in other teleosts [49,50,51,52,53,54]. The growth of *Veraper moseri* increased under the green light spectrum and decreased under the red light spectrum [48]. The growth performance of juvenile *Lateolabrax maculatus* was enhanced under blue, red, and green light conditions compared with white light, with the highest weight gain rate and specific growth rate observed under blue light [51]. The growth of female *Paralichthys olivaceus* increased under 500 nm (green) LED light spectrum [52]. However, previous research indicated that different fish species exhibited varying growth performance under a specific light spectrum during the larval stage. As demonstrated by [25], *H*. *discus hannai* exhibited enhanced growth performance and feed conversion efficiency in the red light spectrum in comparison to the green light spectrum. Previous studies have shown that variation in spectrum preferences among teleosts is primarily attributed to species-specific ecological adaptations. *T. rubripes* inhabits coastal and estuarine environments, where water turbidity often attenuates shorter wavelengths and shifts the available spectrum toward green–yellow regions [3]. This may explain why the green and yellow light spectrum promoted growth and digestive efficiency in our study as these wavelengths likely match the visual environment in which the species evolved. In contrast, species such as *G. morhua* and *S. maximus* exhibit better growth under blue–green light [7]. These species occupy clearer or deeper waters where shorter wavelengths penetrate more effectively, and their visual systems are likely adapted to these conditions. Similarly, the preference of *O. mykiss* for the yellow light spectrum may reflect its stream-dwelling habit, in which dissolved organic matter shifts light toward longer wavelengths [20]. It has been reported that exposure to different light spectra can influence endogenous carotenoid levels [55,56,57], and several studies have shown that carotenoids exhibit a strong positive correlation with fish growth [58,59]. Therefore, the evolutionary adaptation of species to specific light spectra may be attributable, at least in part, to the presence and functions of carotenoids. Notably, larvae reared under full-spectrum white light exhibited a significantly lower survival rate despite comparable growth performance to the green and yellow groups. This may be attributed to the broad-spectrum composition of full-spectrum white light. Unlike monochromatic conditions, full-spectrum white light can act as a composite stressor, simultaneously activating multiple photoreceptor systems and potentially disrupting circadian entrainment. Such photic “noise” could elevate allostatic load, reducing robustness from the larval (20 dah) to the early juvenile (80 dah) stages without manifesting in growth metrics. Future studies should assess oxidative stress markers, cortisol levels, and behavioral responses under full-spectrum white light to clarify its physiological impact.

Somatic cell growth and maturation are regulated by the *gh* axis [60]. *Gh* and insulin-like growth factor 1 (*igf-1*) directly or indirectly promote fish growth through the hypothalamus–pituitary–growth hormone pathway [61]. In response to changes in light conditions, the *gh*/*igf-1* axis combines with insulin-like growth factor binding protein, thereby promoting cell proliferation and fish growth [62]. In the present study, the expression of *gh* in the brain of larvae reared under yellow and green light spectrum conditions was significantly higher than that of other groups at 80 dah. Therefore, high levels of *gh* may promote *T. rubripes* larvae growth in these two groups. Similar results were also reported in other teleost. For example, in *P. olivaceus*, the level of *gh* was higher when fish were reared under the green light spectrum than white light [63]. In *Amphiprion clarkii,* the level of *gh* increased when fish were reared under the green light spectrum, but decreased under the red light spectrum [64]. *Trypsinogen* and *pepsinogen* are the most important digestive enzymes in the gastrointestinal tract of vertebrates. Previous studies have demonstrated its close association with gastric development and digestive function in fish [65]. The digestive process is crucial for converting the food fish eat into usable nutrients that support growth, development, and overall biological functions. As one of the key components in the digestion process, the digestive enzyme activities are influenced by a variety of reasons, such as species specificity [66] and environmental factors [67,68,69]. The findings of this study demonstrate that there is a significant increase in the expression levels of *trypsinogen* in the yellow group and *pepsinogen* in green light spectrum group when compared to other spectral treatments. This may also be one of the reason why *T. rubripes* larvae grow better under yellow and green light conditions. At the same time, previous reports indicated that the digestive capacity of abalone exposed to red light was enhanced, as evidenced by a significant increase in α-amylase concentration and an increase in the intestinal villi surface area. The appetite-related genes (*npy* and *npyr*) were similarly upregulated with red light treatment compared to other LED spectral treatments [70]. This may be the reason for the different expression levels of digestive enzymes under different spectrum exposures.

### 4.2. Effects of Light Spectrum on Retinal Development and Visual Gene Expression

Furthermore, the RPE/TT ratio of *T. rubripes* larvae exposed to the blue light spectrum was significantly lower than green and yellow light treatments. A statistically significant decrease in the PRos/is/TT ratio of larvae exposed to the blue light spectrum was noted in comparison to alternative light treatments. This finding indicated that the retina of *T. rubripes* larvae is more vulnerable to damage from irradiation of the blue light spectrum. Previous studies have shown that fish can regulate the expression of melanin or photoreceptor cells [71]. In teleosts, the melanocortin receptor is regarded as a pivotal regulator of pigment deposition. This function is thought to be achieved by means of a G protein-coupled receptor (*mchr*), which in turn regulates the aggregation of pigment particles in pigment cells. In the retina, melanin-concentrating hormone is a byproduct of melanin, forming the melanin granular layer [72]. Previous studies have shown that *C. auratus* develops retinal melanin shields to protect against the damage of the blue light spectrum [73]. In our experiment, the expression of *mchr1* in the full-spectrum white, blue, and yellow spectra groups was significantly lower than that in green and red light groups. Similarly, the expression level of *mchr2* was significantly lower in the blue light spectrum treatment group compared to the yellow light spectrum. These findings indicate that *T. rubripes* reared under green and red light spectra may protect the retina from damage by increasing the expression of *mchr1*, whereas rearing under yellow light may protect the retina from damage through increased *mchr2* expression. This may suggest the blue light treatment group is more vulnerable to damage because it cannot form melanin granular layer. *Caspase3* is widely used as a key indicator of apoptosis, and it plays a central role in cell apoptosis [74]. In this experiment, the expression of *caspase3* in the blue light treatment group was significantly higher than that in the full-spectrum white light treatment group. Moreover, the thickness of the RPE layer of larvae is reduced when exposed to blue light. Thus, we conclude that melanin migration does not occur in the retina of larvae under blue light, and the retina of *T. rubripes* exhibited retinal thinning and increased apoptotic signaling. This result is consistent with previous research on *G. morhua* and *Salmon salar* [75]. In addition, excessive exposure of the mammalian retina to blue light tends to cause ROS accumulation and oxidative stress in mammals, which affects the structure and function of the retinal mitochondria and triggered mitochondria-involved death signaling pathways. Further verification is required to establish whether this condition occurs in fish [76].

### 4.3. Effects of Light Spectrum on Circadian Rhythm Regulation

Fish usually exhibit circadian rhythms, and light has a significant impact on circadian rhythms, which are controlled by clock genes. Additionally, circadian rhythms are controlled regularly by repeated biochemical, behavioral, and physiological functions [36,41]. This is regulated by the transcription and translation feedback loops of clock genes, which oscillate during a period of approximately 24 h [37,40]. The feedback loop is driven by the heterodimerization of clock and *bmal1* in the nucleus, which regulates the transcription of clock-controlled genes and encodes cyclin (*per*) and cryptochrome (*cry*). In short, four major clock proteins control circadian rhythms. Among them, two clock proteins form a complex to activate the genes of the other two proteins (cyclin *per* and cryptochrome *cry*). After the latter is combined, it produces negative feedback to inhibit the activity of the first pair, thereby weakening its activity and returning to the initial state, allowing the cycle to start again. In our experiment, under red, green, yellow, and full-spectrum white light, the expression levels of *per1a* and *per3* were highest at ZT 0 and lowest at ZT 12, demonstrating a clear diurnal expression pattern. However, in the blue light group, the expression level of *per3* was only highest at ZT 12 and significantly higher than that in the other three groups. Additionally, the expression level of *per1a* at ZT 12 was also highest and significantly higher than the ZT 6 group. Under red, green, yellow, and full-spectrum white light, the expression levels of *cry2* at ZT 6 and ZT 12 were significantly higher than at ZT 0 and ZT 12, indicating a regular diurnal expression pattern. Under blue light, the expression level of the *cry2* gene at ZT 12 was significantly higher than that in other treatment groups. In the blue light group, there was no apparent regularity in the expression of *per3*, *per1a*, and *cry2*, and their diurnal expression patterns differed from the other light groups. Therefore, it may indicate that blue light has a detrimental effect on the circadian rhythms of *T. rubripes* larvae. Previous studies have indicated that the larvae reared under blue light exhibit earlier onset of daily activity rhythms compared to those reared under red or full-spectrum white light in *D. rerio* [77]. Jung et al. (2016) reported that the per mRNA expression level in *C. auratus* increased significantly under green light [78]. In *S. aurata*, the expression of clock genes (*per3*, *cry1*) in larvae fish is affected by the photoperiod [79]. It has also been reported that the *cry1* mRNA and plasma levels of starved *P. olivaceus* were significantly increased under green and blue light. In mammals, blue light could disturb circadian rhythms by interfering with the clock gene in the suprachiasmatic nucleus (SCN), and it is possible that suppression of blue light ameliorates metabolic abnormalities by controlling circadian rhythms [80]. In summary, these results indicate the critical role of light conditions in early teleost development and suggest that light-sensitive molecular clocks exist and function during fish development. However, the molecular mechanisms underlying the effects of the light spectrum on the circadian expression of clock genes in fish remain unclear.

### 4.4. Practical Implications and Future Directions

The findings suggest that optimizing the light spectrum, specifically prioritizing green or yellow spectra while avoiding prolonged blue light exposure, can improve larval rearing efficiency and welfare in *T. rubripes* aquaculture. Several limitations should be considered. First, our assessment of digestive capacity was based on mRNA expression. Although upregulation of these genes under green and yellow light suggests enhanced digestive potential, direct measurements of corresponding protease activities, feed intake rates, or nutrient absorption efficiency are important. Furthermore, light intensity and photoperiod were fixed in the present study, whereas, in nature, they interact dynamically with the spectrum. Our findings at a single intensity and photoperiod may not fully replicate wild conditions. In addition, at the same power, the photon flux density, measured in μmol m^−2^ s^−1^, differs between spectrum. Consequently, the observed differences in growth and physiology may be caused not by light quality (spectrum) alone but by differences in photon quantity per unit area and time. Addressing these limitations using integrated measurements of digestive enzyme activities, feed utilization efficiency, and factorial combinations of light spectrum, intensity, and photoperiod represents an important direction for future research. Future studies should also assess oxidative stress markers, cortisol levels, and behavioral responses under full-spectrum white light to clarify its physiological impact.

## 5. Conclusions

This study demonstrated that the light spectrum significantly influences growth, visual development, and circadian regulation in *T. rubripes* larvae. Green and yellow light spectra promoted growth performance; enhanced the expression of *gh*, *trypsinogen*, and *pepsinogen*; and supported efficient digestive function. In contrast, blue light exposure led to retinal thinning, increased *caspase3* expression, and disrupted circadian expression patterns of clock genes (*per3*, *per1a*, *cry2*), indicating adverse effects on visual health and biological rhythms. Based on our study, we propose the following practical lighting strategies for *T. rubripes* larviculture: (1) To promote growth and digestive efficiency, the use of green (525–530 nm) or yellow (590–595 nm) LED light is recommended throughout the larval stage (e.g., from hatch to 80 dah). (2) To safeguard retinal development and circadian function, prolonged, direct exposure to narrow-band blue light (450–455 nm) should be avoided. These recommendations are based on the significant physiological responses observed under controlled, monochromatic conditions and provide a targeted starting point for optimizing light environments for commercial *T. rubripes* aquaculture.

## Figures and Tables

**Table 1:** Primers for qPCR in this study.

Gene Name	Sequence of Primer	Amplification Efficiency (%)	Product Length (bp)
*ef1*α (AB193485.1)	F: AGGAGGGCAATGCTAGTGG	100.6	204
	R: TGGTCAGGTTGACGGGAG		
*β-actin* (XM_003964421.2)	F:AACCAAATGCCCAACAACTTC	99.3	213
	R: GATCCCCAGATGCAACAGAAC		
*gh* (XM_003968318)	F: ACAAGCACGAGACACAAC	93.8	193
	R: AGCAGAACCTCCAGACAG		
*trypsinogen* (NC_051055.1)	F: ATCGTCGGAGGGTATGAGTG	98.0	362
	R: CATCCAGAGACTGTGCACATG		
*pepsinogen* (NC_058061.1)	F: TGAGTCCATGACTAATGATGCT	95.1	292
	R: CACCTGATTGGCCACAGAG		
*mchr1* (NC_000081.7)	F: GCAAACGGACAGAAGCATTT	99.3	183
	R: GCGGTAGAGGAAGACCCTTT		
*mchr2* (NC_052151.1)	F: TCACCGCAAAAGGAAGTAGC	99.9	216
	R: CACTCAGGGCTGAAGTTGC		
*caspase3* (NM_001032699.1)	F: CGAGGGCGTGTTTTTTGGT	103.3	262
	R: GGGATCTTGGTGGTGCTGC		
*rh1* (NM_001033849.1)	F: GAACTACGTCCTGCTCAACCTG	102.6	148
	R: CCCTCCTAAAGTGGCAAAGAAT		
*rh2* (NM_001033712.1)	F: GCAGTCAAACGATTCCCAT	105.3	190
	R: GTTCCTCTAACAACCACCAAAA		
*sws2* (XM_003973672.3)	F: GGGACACCATTTGATCTGAGAC	93.8	106
	R: AGCGGAACTGTTTATTGAGGAC		
*lws* (XM_003973673.3)	F: CAATGTGCGTCTTTGAGGGT	95.9	231
	R: TCTTCAGTCCATGAGGCCAG		
*per3* (ENSTRUT00000007589)	F: GCGTTAAACAAGTCCAAGCTAACA	101.9	147
	R: CACCACAAAGGAGTCCGTGTT		
*per1a* (ENSTRUT00000037001)	F: CACCCTCAACGCACTCAAATA	96.3	157
	R: GTACTCCGAGGTGATGTTGTCC		
*cry2* (ENSTRUG00000015322)	F: GTCAACAGGTGGCGGTTTCT	96.9	124
	R: CCGTGAGATCTTCCATTCCTTAAA		

**Table 2:** Growth indexes of *Takifugu rubripes* larvae under different spectral conditions (n = 3; ±SD).

Growth Index	Light Spectrum Treatment Groups														
	20 dah					60 dah					80 dah				
	B	R	G	Y	W	B	R	G	Y	W	B	R	G	Y	W
Body length (mm)	5.7211 ± 1.686 × 10^−1 a^	6.48 ± 1.501 × 10^−1 b^	6.435 ± 3.458 × 10^−1 b^	6.515 ± 1.686 × 10^−1 b^	6.0105 ± 1.652 × 10^−1 ab^	32.9833 ± 5.313 × 10^−1 a^	35.3533 ± 1.3249 ^ab^	39.9167 ± 5.572 × 10^−1 c^	36.18 ± 8.969 × 10^−1 b^	32.8333 ± 6.101 × 10^−1 a^	48.8833 ± 1.2428 ^a^	49.1367 ± 3.813 × 10^−1 a^	55.6967 ± 9.743 × 10^−1 b^	55.3733 ± 1.4716 ^b^	53.1533 ± 9.969 × 10^−1 b^
Total length (mm)	7.5789 ± 2.348 × 10^−1 a^	8.19 ± 4.965 × 10^−1 a^	8.28 ± 50,098 × 10^−1 a^	8.7 ± 4.705 × 10^−1 a^	7.7737 ± 20,805 × 10^−1 a^	38.88 ± 4.293 × 10^−1 a^	42.26 ± 1.6082 ^b^	47.71 ± 8.854 × 10^−1 c^	42.2767 ± 9.787 × 10^−1 b^	40.8667 ± 2.522 × 10^−1 ab^	54.4833 ± 1.3869 ^a^	55.1467 ± 6.68 × 10^−1 a^	63.37 ± 1.00724 ^b^	62.1033 ± 1.7991 ^b^	60.8367 ± 1.6189 ^b^
Eye diameter (mm)	7.658 × 10^−1^ ± 1.81 × 10^−2 ab^	7.355 × 10^−1^ ± 1.29 × 10^−2 a^	6.87 × 10^−1^ ± 3.23 × 10^−2 a^	8.45 × 10^−1^ ± 3.41 × 10^−2 a^	7.358 × 10^−1^ ± 2.17 × 10^−2 a^	3.57 ± 3.51 × 10^−2 b^	3.7233 ± 1.053 × 10^−1 bc^	3.7867 ± 1.2 × 10^−2 c^	3.6133 ± 3.53 × 10^−2 bc^	3.2333 ± 5.89 × 10^−2 a^	4.6267 ± 6.64 × 10^−2 a^	4.61 ± 6.51 × 10^−2 a^	4.77 ± 1.405 × 10^−1 a^	4.8133 ± 8.41 × 10^−2 a^	4.8 ± 2.08 × 10^−2 a^
Wet weight (g)	1.33 × 10^−2^ ± 1.1 × 10^−4 a^	1.59 × 10^−2^ ± 5.4 × 10^−4 b^	1.63 × 10^−2^ ± 2.3 × 10^−4 b^	2.14 × 10^−2^ ±4.8 × 10^−4 c^	1.39 × 10^−2^ ±2.1 × 10^−4 a^	1.6122 ± 5.718 × 10^−1 a^	2.0929 ± 1.669 × 10^−1 b^	2.6761 ± 1.377 × 10^−1 c^	1.9642 ± 8.84 × 10^−2 ab^	1.9338 ± 3.59 × 10^−2 ab^	4.3199 ± 3.154 × 10^−1 a^	4.2915 ± 2.06 × 10^−2 a^	6.3124 ± 2.114 × 10^−1 b^	6.1749 ± 6.151 × 10^−1 b^	6.0857 ± 2.089 × 10^−1 b^

Different lowercase letters indicate significant differences between each treatment group (one-way ANOVA, *p* < 0.05, n = 3 tanks per group). Groups sharing the same letter are not significantly different. Blue light spectrum (B), red light spectrum (R), green light spectrum (G), yellow light spectrum (Y), and full-spectrum white (W) light.

## Data Availability

The datasets generated and analyzed during the current study are not publicly available due to subsequent research, but are available from the corresponding author on reasonable request.

## References

[1] Niven J.E., Laughlin S.B. (2008). Energy Limitation as a Selective Pressure on the Evolution of Sensory Systems. J. Exp. Biol..

[2] Hagemann A., Vorren S.H., Attramadal Y., Evjemo J.O., Olsen Y. (2017). Effects of Different Wavelengths and Intensities of Visible Light on the Hatching Success of Acartia Tonsa Dana Eggs. Aquac. Int..

[3] Chung S.P., Gardner W.D., Landry M.R., Richardson M.J., Walsh I.D. (1998). Beam Attenuation by Microorganisms and Detrital Particles in the Equatorial Pacific. J. Geophys. Res. Ocean..

[4] Pitt K.A., Connolly R.M., Meziane T. (2009). Stable Isotope and Fatty Acid Tracers in Energy and Nutrient Studies of Jellyfish: A Review. Hydrobiologia.

[5] Zhang Z., Fei F., Wang L., Rao Y., Li W., Gao X., Li A., Liu B. (2026). A Review of Adaptive Mechanisms in Fish Retinal Structure and Opsins Under Light Environment Regulation. Fishes.

[6] Ruchin A. (2018). Light Spectrum Impacts on Early Development of Amphibians (Amphibia: Anura and Caudata). Pertanika J. Trop. Agric. Sci..

[7] Sierra-Flores R., Davie A., Grant B., Carboni S., Atack T., Migaud H. (2016). Effects of Light Spectrum and Tank Background Colour on Atlantic Cod (*Gadus morhua*) and Turbot (*Scophthalmus maximus*) Larvae Performances. Aquaculture.

[8] Wu L., Wang Y., Han M., Song Z., Song C., Xu S., Li J., Wang Y., Li X., Yue X. (2020). Growth, Stress and Non-Specific Immune Responses of Turbot (*Scophthalmus maximus*) Larvae Exposed to Different Light Spectra. Aquaculture.

[9] Reddy S.R., Kote G. (1975). Predatory Behavior of Gambusia Affinis in Relation to Different Light Colors. Physiol. Behav..

[10] Volpato G.L., Duarte C.R.A., Luchiari A.C. (2004). Environmental Color Affects Nile Tilapia Reproduction. Braz. J. Med. Biol. Res..

[11] Luchiari A.C., do Amaral Duarte C.R., de Morais Freire F.A., Nissinen K. (2007). Hierarchical Status and Colour Preference in Nile Tilapia (*Oreochromis niloticus*). J. Ethol..

[12] Höglund E., Balm P.H.M., Winberg S. (2002). Behavioural and Neuroendocrine Effects of Environmental Background Colour and Social Interaction in Arctic Charr (*Salvelinus alpinus*). J. Exp. Biol..

[13] Timmer R., Magellan K. (2011). The Effects of Light Intensity and Color on Aggressive Interactions in the Dusky Kob, *Argyrosomus japonicus*. Isr. J. Aquac. Bamidgeh.

[14] Hailman J.P., Jaeger R.G. (1974). Phototactic Responses to Spectrally Dominant Stimuli and Use of Colour Vision by Adult Anuran Amphibians: A Comparative Survey. Anim. Behav..

[15] Yeh N., Yeh P., Shih N., Byadgi O., Chih Cheng T. (2014). Applications of Light-Emitting Diodes in Researches Conducted in Aquatic Environment. Renew. Sustain. Energy Rev..

[16] Migaud H., Cowan M., Taylor J., Ferguson H.W. (2007). The Effect of Spectral Composition and Light Intensity on Melatonin, Stress and Retinal Damage in Post-Smolt Atlantic Salmon, *Salmo salar*. Aquaculture.

[17] Noureldin S.M., Diab A.M., Salah A.S., Mohamed R.A. (2021). Effect of Different Monochromatic LED Light Colors on Growth Performance, Behavior, Immune-Physiological Responses of Gold Fish, *Carassius auratus*. Aquaculture.

[18] Villamizar N., García-Alcazar A., Sánchez-Vázquez F.J. (2009). Effect of Light Spectrum and Photoperiod on the Growth, Development and Survival of European Sea Bass (*Dicentrarchus labrax*) Larvae. Aquaculture.

[19] Choi C.Y., Shin H.S., Choi Y.J., Kim N.N., Lee J., Kil G.-S. (2012). Effect of LED Light Spectra on Starvation-Induced Oxidative Stress in the Cinnamon Clownfish *Amphiprion melanopus*. Comp. Biochem. Physiol. Part A Mol. Integr. Physiol..

[20] Heydarnejad M.S., Parto M., Pilevarian A.A. (2013). Influence of Light Colours on Growth and Stress Response of Rainbow Trout (*Oncorhynchus mykiss*) under Laboratory Conditions. J. Anim. Physiol. Anim. Nutr..

[21] Heydarnejad M.S., Fattollahi M., Khoshkam M. (2017). Influence of Light Colours on Growth and Stress Response of Pearl Gourami Trichopodus Leerii under Laboratory Conditions. J. Ichthyol..

[22] Liu S., Fang Y., Liu Y., Li X., Sun F., Wu Y., Ma Z., Ma H. (2022). Effects of Different LED Spectra on Growth and Expression of GH/IGF-I Axis and Apoptosis Related Genes in Juvenile *Takifugu rubripes*. Front. Mar. Sci..

[23] Kim N.N., Choi Y.J., Shin H.S., Lim J.R., Han J.M., Cho J.H., Lee J., Kil G.-S., Choi C.Y. (2014). The Effect of LED Light Spectra on Antioxidant System by Thermal Stress in Goldfish, *Carassius auratus*. Mol. Cell. Toxicol..

[24] Lopez-Betancur D., Moreno I., Guerrero-Mendez C., Gómez-Meléndez D., Macias P.M.d.J., Olvera-Olvera C. (2020). Effects of Colored Light on Growth and Nutritional Composition of Tilapia, and Biofloc as a Food Source. Appl. Sci..

[25] Gao X., Pang G., Luo X., You W., Ke C. (2021). Effects of Light Cycle on Motion Behaviour and Melatonin Secretion in *Haliotis discus hannai*. Aquaculture.

[26] Blier P., Pelletier D. (1997). Does Aerobic Capacity Set a Limit on Fish Growth Rate?. Rev. Fish. Sci..

[27] García-Gasca A., Galaviz M.A., Gutiérrez J.N., García-Ortega A. (2006). Development of the Digestive Tract, Trypsin Activity and Gene Expression in Eggs and Larvae of the Bullseye Puffer Fish *Sphoeroides annulatus*. Aquaculture.

[28] Durigon E.G., Almeida A.P.G., Jerônimo G.T., Baldisserotto B., Emerenciano M.G.C. (2019). Digestive Enzymes and Parasitology of Nile Tilapia Juveniles Raised in Brackish Biofloc Water and Fed with Different Digestible Protein and Digestible Energy Levels. Aquaculture.

[29] Xue Y., Zhao J., Deng Y., Wu X., Miao W. (2013). Cloning and Spatiotemporal Expression of Pepsinogen and Gastric Proton Pump Genes from Mandarin Fish (*Siniperca chuatsi*) during Early Ontogeny. Fish Physiol. Biochem..

[30] Nazemroaya S., Yazdanparast R., Nematollahi M.A., Farahmand H., Mirzadeh Q. (2015). Ontogenetic Development of Digestive Enzymes in Sobaity Sea Bream Sparidentex Hasta Larvae under Culture Condition. Aquaculture.

[31] Furné M., Hidalgo M.C., López A., García-Gallego M., Morales A.E., Domezain A., Domezainé J., Sanz A. (2005). Digestive Enzyme Activities in Adriatic Sturgeon Acipenser Naccarii and Rainbow Trout *Oncorhynchus mykiss*. A Comparative Study. Aquaculture.

[32] Kim S.-C., Moon J.-S., Cadangin J., Lee E.-S., Joo B.-H., Kim H.-S., Hur S.-W., Choi Y.H. (2025). Effect of LED Spectrum on the Vision, Appetite, and Growth of Red Seabream (*Pagrus major*). Aquaculture.

[33] Shand J., Davies W.L., Thomas N., Balmer L., Cowing J.A., Pointer M., Carvalho L.S., Trezise A.E.O., Collin S.P., Beazley L.D. (2008). The Influence of Ontogeny and Light Environment on the Expression of Visual Pigment Opsins in the Retina of the Black Bream, *Acanthopagrus butcheri*. J. Exp. Biol..

[34] Wagner H.-J., Kröger R.H.H. (2005). Adaptive Plasticity during the Development of Colour Vision. Prog. Retin. Eye Res..

[35] DeBose J.L., Lema S.C., Nevitt G.A. (2008). Dimethylsulfoniopropionate as a Foraging Cue for Reef Fishes. Science.

[36] King D.P., Takahashi J.S. (2000). Molecular Genetics of Circadian Rhythms in Mammals. Annu. Rev. Neurosci..

[37] Simensen L.M., Jonassen T.M., Imsland A.K., Stefansson S.O. (2000). Photoperiod Regulation of Growth of Juvenile Atlantic Halibut (*Hippoglossus hippoglossus* L.). Aquaculture.

[38] Klein D.C. (2007). Arylalkylamine N-Acetyltransferase: “The Timezyme”. J. Biol. Chem..

[39] Delaunay F., Thisse C., Marchand O., Laudet V., Thisse B. (2000). An Inherited Functional Circadian Clock in Zebrafish Embryos. Science.

[40] Besharse J.C., Zhuang M., Freeman K., Fogerty J. (2004). Regulation of Photoreceptor Per1 and Per2 by Light, Dopamine and a Circadian Clock. Eur. J. Neurosci..

[41] Pierce L.X., Noche R.R., Ponomareva O., Chang C., Liang J.O. (2008). Novel Functions for Period 3 and Exo-Rhodopsin in Rhythmic Transcription and Melatonin Biosynthesis within the Zebrafish Pineal Organ. Brain Res..

[42] Choi J.Y., Kim T.H., Choi Y.J., Kim N.N., Oh S.-Y., Choi C.Y. (2016). Effects of Various LED Light Spectra on Antioxidant and Immune Response in Juvenile Rock Bream, Oplegnathus Fasciatus Exposed to Bisphenol A. Environ. Toxicol. Pharmacol..

[43] Chang C.-H., Yan H.Y. (2019). Plasticity of Opsin Gene Expression in the Adult Red Shiner (*Cyprinella lutrensis*) in Response to Turbid Habitats. PLoS ONE.

[44] Li X., Liu S., Fan K., Zhang J., Wei P., Liu Y., Tian Y., Ma H. (2022). Effects of Illumination Intensities on Growth, Digestive and Metabolic Enzyme Activities and Antioxidant Capacities of Juvenile *Takifugu rubripes*. Aquaculture.

[45] Liu Z., Wang X., Ma A., Zhu L., Chang H., Sun Z. (2022). Construction of a High-Density Genetic Linkage Map and QTL Mapping of Growth and Cold Tolerance Traits in Tiger Puffer *Takifugu rubripes*. Aquaculture.

[46] Li X., Chang L., Han F., Li X., Xiao L., Huang E., Yang Y., Su L., Pang S. (2025). Challenges of Genetic Homogeneity in Aquaculture of the Kelp Saccharina Japonica: Insights from China in Ten Year’s Retrospect. Aquac. Rep..

[47] Xia Y., Hua X., Zhu S., Liu Y., Liu P. (2021). Characterization and Expression Analysis of the Complement Component 8β from Pufferfish (*Takifugu rubripes*). Aquac. Rep..

[48] Yamanome T., Mizusawa K., Hasegawa E., Takahashi A. (2009). Green Light Stimulates Somatic Growth in the Barfin Flounder *Verasper moseri*. J. Exp. Zool. Part A Ecol. Genet. Physiol..

[49] Sun X., Zhao X., Li L., Gao Q., Dong S. (2022). Growth, Oxidative Stress and the Non-Specific Immune Response of *Oncorhynchus mykiss* Exposed to Different Light Spectra. Aquac. Res..

[50] Zheng J.-L., Gao L., Zhang H.-T., Chen X., Zhu Q.-L., Han T. (2024). LED Light Spectra Differently Affected Growth, Antioxidant Capacity, Stress Response, GH/IGF and HPI Axis in Largemouth Bass (*Micropterus salmoides*) Larvae. Aquaculture.

[51] Zheng J., Ma B., Jiang Y., Cui A., Xu Y., Cai X., Wang B., Jiao K., Li T., Liu H. (2026). Effects of Different Monochromatic Light Colours on Growth Performance, Feeding, Antioxidant Capacity, Transcriptomic Response and Gut Microbiota of Juvenile Spotted Sea Bass (*Lateolabrax maculatus*). Aquaculture.

[52] Hettiarachchi S.A., Hyeon J.-Y., Byun J.-H., Kim B.-H., Lee K., Park J.-E., Choi S.-Y., Yoon Y.S., Oh C., Hur S.-P. (2025). Growth Enhancement and Gonadal Development Disruption Using 500-Nm LED Light in Commercial-Scale Olive Flounder (*Paralichthys olivaceus*) Aquaculture. Aquac. Int..

[53] Li W., Zhang Z., Liu B., Fang Y., Cao S., Li W., Sun Y., He C., Zhang C., Fei F. (2025). Effects of Different Light Spectra on Oxidative Stress and Nutritional Quality of the Fish *Plectropomus leopardus*. Fishes.

[54] Muller R.L., Cerdeira Lopes A.C., de Mattos B.O., Sánchez-Vázquez F.J., Carvalho T.B. (2025). Light Wavelength Affects Ontogeny, Survival and Intraspecific Predation in Larvae of Matrinxã, Brycon Amazonicus. J. Fish Biol..

[55] Ai Z., Yang Z., Ming J., Zhang L., Chen X., Xu Z., Zhang W., Wang A., Tian H., Xia S. (2026). Effects of Four Light Colors on Physiology, Antioxidant Enzyme Activity, Shell Pigmentation, and Genes Associated with Body Color Formation in *Procambarus clarkii*. Fishes.

[56] Pavlidis M., Karkana M., Fanouraki E., Papandroulakis N. (2008). Environmental Control of Skin Colour in the Red Porgy, *Pagrus pagrus*. Aquac. Res..

[57] Dong M., Zhang Y., Yu Q., Liu Q., Zhou X., Jin C. (2023). Regulation of Light Quality on Lipid Production, Biodiesel Quality, and Nutritional Quality of *Phaeodactylum tricornutum*. Aquac. Int..

[58] Tarek M., Rana S., Himel I.A., Sunny Z.A., Khan M.S., Shakib I.A., Tushar M.T.H., Shimul S.A., Nahid S.A.A. (2026). Effect of Dietary Astaxanthin on Growth Performance, Feed Utilisation, Body Colouration and Survival in Nile Tilapia (*Oreochromis niloticus*). Aquac. Fish Fish..

[59] Xue Y., Wang Z., Liu M., Yi G., Huang X., Wang W. (2025). From Indicator Evaluation to Optimization Decision: Effects of Synthetic vs. Natural Astaxanthin on Pigmentation, Growth, and Health in *Penaeus vannamei*. Aquaculture.

[60] Ayres J.S. (2020). The Biology of Physiological Health. Cell.

[61] Mommsen T.P. (2001). Paradigms of Growth in Fish. Comp. Biochem. Physiol. Part B Biochem. Mol. Biol..

[62] Frank S.J. (2020). Classical and Novel GH Receptor Signaling Pathways. Mol. Cell. Endocrinol..

[63] Zou Y., Peng Z., Wang W., Liang S., Song C., Wang L., Wu Z., Wu Q., Tan X., You F. (2022). The Stimulation Effects of Green Light on the Growth, Testicular Development and Stress of Olive Flounder *Paralichthys olivaceus*. Aquaculture.

[64] Shin H.S., Lee J., Choi C.Y. (2012). Effects of LED Light Spectra on the Growth of the Yellowtail Clownfish *Amphiprion clarkii*. Fish. Sci..

[65] Gottlieb Almeida A.P., Zardo E.L., Toni C., Behr E.R., Picolli da Silva L., Vieira J.P., Loro V.L., Baldisserotto B. (2018). Composition of Gastrointestinal Content, Protease and Lipase Activities in Summer and Winter of Four Freshwater Siluriforms (Teleostei: Actinopterygii) with Two Different Feeding Habits. Zoologia.

[66] Chan A.S., Horn M.H., Dickson K.A., Gawlicka A. (2004). Digestive Enzyme Activities in Carnivores and Herbivores: Comparisons among Four Closely Related Prickleback Fishes (Teleostei: Stichaeidae) from a California Rocky Intertidal Habitat. J. Fish Biol..

[67] Psochiou E., Sarropoulou E., Mamuris Z., Moutou K.A. (2007). Sequence Analysis and Tissue Expression Pattern of *Sparus aurata* Chymotrypsinogens and Trypsinogen. Comp. Biochem. Physiol. Part B Biochem. Mol. Biol..

[68] Nikolopoulou D., Moutou K.A., Fountoulaki E., Venou B., Adamidou S., Alexis M.N. (2011). Patterns of Gastric Evacuation, Digesta Characteristics and pH Changes along the Gastrointestinal Tract of Gilthead Sea Bream (*Sparus aurata* L.) and European Sea Bass (*Dicentrarchus labrax* L.). Comp. Biochem. Physiol. Part A Mol. Integr. Physiol..

[69] Pujante I., Moyano F., Martos-Sitcha J., Mancera J., Martínez-Rodríguez G. (2018). Effect of Different Salinities on Gene Expression and Activity of Digestive Enzymes in the Thick-Lipped Grey Mullet (*Chelon labrosus*). Fish Physiol. Biochem..

[70] Joo B.-H., Cadangin J., Lee C.-H., Lee E.-S., Moon J.-S., Hur S.-W., Min B.H., Nam T.-J., Choi Y.-H. (2026). Optimizing LED Light Spectra for Juvenile Abalone Aquaculture: Effects on Growth, Digestion, Immunity, Retinal Structure, Meat Textural Property and Sensory Profile. Aquaculture.

[71] Allison W.T., Hallows T.E., Johnson T., Hawryshyn C.W., Allen D.M. (2006). Photic History Modifies Susceptibility to Retinal Damage in Albino Trout. Vis. Neurosci..

[72] Rance T., Baker B.I. (1979). The Teleost Melanin-Concentrating Hormone—A Pituitary Hormone of Hypothalamic Origin. Gen. Comp. Endocrinol..

[73] Song J.A., Kim N.N., Choi Y.J., Choi C.Y. (2016). Effect of Green Light Spectra on the Reduction of Retinal Damage and Stress in Goldfish, *Carassius auratus*. Biochem. Biophys. Res. Commun..

[74] Häcker G. (2000). The Morphology of Apoptosis. Cell Tissue Res..

[75] Vera L.M., Migaud H. (2009). Continuous High Light Intensity Can Induce Retinal Degeneration in Atlantic Salmon, Atlantic Cod and European Sea Bass. Aquaculture.

[76] Tao J.-X., Zhou W.-C., Zhu X.-G. (2019). Mitochondria as Potential Targets and Initiators of the Blue Light Hazard to the Retina. Oxid. Med. Cell Longev..

[77] Di Rosa V., Frigato E., López-Olmeda J.F., Sánchez-Vázquez F.J., Bertolucci C. (2015). The Light Wavelength Affects the Ontogeny of Clock Gene Expression and Activity Rhythms in Zebrafish Larvae. PLoS ONE.

[78] Jung S.J., Choi Y.J., Kim N.N., Choi J.Y., Kim B.-S., Choi C.Y. (2016). Effects of Melatonin Injection or Green-Wavelength LED Light on the Antioxidant System in Goldfish (*Carassius auratus*) during Thermal Stress. Fish Shellfish. Immunol..

[79] Mata-Sotres J.A., Martínez-Rodríguez G., Pérez-Sánchez J., Sánchez-Vázquez F.J., Yúfera M. (2015). Daily Rhythms of Clock Gene Expression and Feeding Behavior during the Larval Development in Gilthead Seabream, *Sparus aurata*. Chronobiol. Int..

[80] Nagai N., Ayaki M., Yanagawa T., Hattori A., Negishi K., Mori T., Nakamura T.J., Tsubota K. (2019). Suppression of Blue Light at Night Ameliorates Metabolic Abnormalities by Controlling Circadian Rhythms. Investig. Ophthalmol. Vis. Sci..

